# Monospecific antibody targeting of CDH11 inhibits epithelial-to-mesenchymal transition and represses cancer stem cell-like phenotype by up-regulating miR-335 in metastatic breast cancer, in vitro and in vivo

**DOI:** 10.1186/s12885-019-5811-1

**Published:** 2019-06-27

**Authors:** Jia-Hong Chen, Wen-Chien Huang, Oluwaseun Adebayo Bamodu, Peter Mu-Hsin Chang, Tsu-Yi Chao, Tse-Hung Huang

**Affiliations:** 1 Graduate Institute of Clinical Medicine, College of Medicine, Taipei Medical University, Taipei City, 110 Taiwan; 2 Division of Hematology/Oncology, Department of Medicine, Tri-Service General Hospital, National Defence Medical Center, Taipei City, 114 Taiwan; 3 Division of Thoracic Surgery, Department of Surgery, MacKay Memorial Hospital, Taipei, Taiwan; 4 MacKay Medical College, Taipei City, 252 Taiwan; 5 Department of Hematology and Oncology, Cancer Center, Taipei Medical University-Shuang Ho Hospital, New Taipei City, 235 Taiwan; 6 Department of Medical Research and Education, Taipei Medical University-Shuang Ho Hospital, New Taipei City, 235 Taiwan; 7 Department of Oncology, Taipei Veterans General Hospital, Taipei City, 112 Taiwan; 8 Faculty of Medicine, National Yang Ming University, Taipei, 112 Taiwan; 9 Department of Traditional Chinese Medicine, Chang Gung Memorial Hospital, Keelung, 105 Taiwan; 10 School of Traditional Chinese Medicine, Chang Gung University, Taoyuan City, 333 Taiwan; 11 School of Nursing, National Taipei University of Nursing and Health Sciences, Taipei City, 112 Taiwan; 12 Taipei Cancer Center, Taipei Medical University, Taipei City, 110 Taiwan

**Keywords:** Invasive breast cancer, Metastasis, Cancer stem cell, CDH11/β-catenin signaling, miR-335, Antibody therapeutics

## Abstract

**Background:**

Metastasis is a leading cause of breast cancer mortality. The induction of epithelial-to-mesenchymal transition (EMT) and complex oncogenic signaling is a vital step in the evolution of highly metastatic and therapeutically-intractable breast cancer; necessitating novel target discovery or development of therapeutics that target metastatic breast cells (MBCs).

**Methods:**

To achieve this, this study employs a combination of in silico bioinformatics analyses, protein and transcript analyses, drug sensitivity assays, functional assays and animal studies.

**Results:**

The present study identified CDH11 as an inductor and/or facilitator of metastatic signaling, and biomarker of poor prognosis in MBCs. Furthermore, we showed that in the presence of CDH11-rich cancer-associated fibroblasts (CAFs), MCF7 and MDA-MB-231 MBC cell lines acquired enhanced metastatic phenotype with increased CDH11, β-catenin, vimentin, and fibronectin (FN) expression. We also demonstrated, for the first time to the best of our knowledge that exposure to anti-CDH11 antibody suppresses metastasis, reduces CDH11, FN and β-catenin expression, and abrogate the cancer stem cell (CSC)-like traits of MBC cells. Interestingly, ectopic expression of miR-335 suppressed CDH11, β-catenin and vimentin expression, in concert with attenuated metastatic and CSC potentials of the MBC cells; conversely, inhibition of miR-335 resulted in increased metastatic potential. Finally, corroborating the *in silica* and in vitro findings, in vivo assays showed that the administration of anti-CDH11 antibody or miR-335 mimic suppressed tumorigenesis and inhibited cancer metastasis.

**Conclusions:**

These findings validate our hypotheses that miR-335 mediates anti-CDH11 antibody therapy response and that an enhanced miR-335/CDH11 ratio elicits marked suppression of the MBC CSC-like and metastatic phenotypes, thus revealing a therapeutically-exploitable inverse correlation between CDH11-enhanced CSC-like and metastatic phenotype and miR-335 expression in MBCs. Thus, we highlight the therapeutic promise of humanized anti-CDH11 antibodies or miR-335-mimic, making a case for their clinical application as efficacious therapeutic option in patients with MBC.

**Electronic supplementary material:**

The online version of this article (10.1186/s12885-019-5811-1) contains supplementary material, which is available to authorized users.

## Background

Tissue invasion and metastasis is one of the hallmarks of cancer [1]. With 2,088,849, new cases, 626,679 deaths, and 5-year prevalence of 6,875,099 globally in 2018 alone [2], invasive breast cancer remains a clinical challenge and continues to bear heavily on global economies. Data from the Taiwan Cancer Registry suggest a rise in breast cancer incidence, with a mean annual age-standardized rate (ASR) of 3.5 per 100,000 person-years (3.1–3.8; *p* < 0.05), and a 10-year annual percentage change (APC) in breast cancer survival of 0.0 (0.0–0.0) amongst patients with advanced stage or metastatic disease, highlighting the great clinical and socio-economic burden of metastatic breast cancer in Taiwan [3]. Against this background, despite advances in diagnostic and therapeutic strategies, and improved understanding of the pathobiology of invasive and/or metastatic breast cancer, there is the cognizance that they can only be managed therapeutically, but not cured, thus, necessitating the discovery of novel therapeutic target(s) or development of more effective metastasis-limiting therapeutic strategies.

Recently, Marino N, et al., in their seminal work provided important insight into breast cancer-relevant metastasis gene signature [4]. Their work suggested the expression of cadherin 11 (CDH11) was inversely correlated with that of suppressors of metastasis in breast cancer. CDH11 (also known as osteoblast (OB)-cadherin), from the cadherin super-family and originally identified in mouse osteoblasts, mediates cell-cell adhesion in a calcium-dependent manner [5]. Detected mainly in mesenchymal but not in epithelial tissues during embryogenesis, CDH11 is a biomarker for mesenchymal phenotype [6], and is associated with cell lines or tissues of aggressive cancers, especially as an alternatively-spliced variant of CDH11 was identified in breast cancer cell lines and shown to promote invasiveness [7, 8]. This documented differential or alternative splicing of a gene connotes its ability to code for several genes, increasing its repertoire of genomic information and post-transcriptional functional capacity. Consistent with contemporary knowledge and in the context of the present study, it is probable that CDH11-rich metastatic breast cells harbor profound alterations in their transcriptome partly by adopting cancer-specific CDH11 splicing variants, and that these variants with their encoded proteins are not passive epiphenomena of the pathogenesis of cancerous breast cells, but rather drive metastasis, and disease progression or actively contribute to specific hallmarks of cancer [9, 10]. This is particular true for CDH11, where the function of its Src-like COOH terminus-truncated variant remains unknown, despite documented co-expression and the implication of this aberrant co-expression in enhanced invasiveness of cancerous cells [7, 9]. Nevertheless, the biological role of CDH11, its associated signaling network(s), and its targetability as an efficacious therapeutic strategy in patients with breast, remains underexplored. Recently we demonstrated that by modulating β-catenin, CDH11 regulates the canonical WNT signalling pathway, and its inhibition suppresses the CSC-like phenotypes and tumor growth of TNBC cells, making a case for the therapeutic targeting of CDH11 as a novel therapeutic approach in TNBC treatment [11]. As a sequela, in the present study, we elucidated on the involvement of CDH11 in breast cancer metastasis, its epigenetic modulation, and its mechanistic undertone.

MicroRNAs (miRNAs, miRs) are small (~ 22 nucleotide long) non-coding RNAs involved in the regulation of gene expression, and implicated in a broad spectrum of diseases, including cancer. MiRNAs regulate the expression of protein-coding genes by binding to the 3′ untranslated region (3′ UTR) of their target messenger RNA (mRNA), consequently degrading the target mRNA through the Dicer RNA-induced silencing complex (RISC). The erstwhile intrinsic balance between various miRs in normal cells is disrupted in cancerous cells, resulting in the induction of oncogenes and oncogenic signals, plus inactivation of tumor suppressor [12, 13]. Many miRNAs have been shown to repress metastasis, including miR-335-5p which was recently shown to decrease the migration, invasion, and anchorage-independent cell growth in gastric cancer [14]. However, the probable role of miR-335 in CDH11-driven metastatic breast cancer remains to be explored. Therefore, the present study by using public cancer databases and analytical tools, consistent with our previous findings [11], demonstrate that elevated CDH11 mRNA level is associated with invasive breast carcinoma as compared to normal breast tissue. A strong positive correlation was also established between the expression CDH11 and metastasis-associated genes, β-catenin and vimentin in MCF7 and MDA-MB-231 cells in the presence of cancer-associated fibroblasts (CAFs), suggesting enhanced metastatic potential. Conversely, the inhibition of CDH11 by anti-CDH11 antibody concomitantly suppressed the expression of CDH11, β-catenin and vimentin and elicited reduced metastatic and CSC-like phenotypes. Mechanistically, an inverse correlation was observed between CDH11 and miR-335 expression level, with exposure to monospecific anti-CDH11 antibody inducing significant increase in the miR-335/CDH11 ratio and markedly suppressed metastatic potential. Using murine tumor xenograft models, we demonstrated in vivo that anti-CDH11 antibody treatment significantly reduces distant metastasis in MCF7 or MDA-MB-231-bearing mice. Collectively, we showed that elevated CDH11 is associated with breast cancer metastatic and CSC-like phenotypes, and its monospecific antibody targeting is a putative effective metastasis-limiting therapeutic option for treatment of metastatic breast cancer.

## Methods

### Cell culture and reagents

Human metastatic breast cancer cell lines, MCF7 (ER+/PR+/HER2−/glucocorticoid receptor (GCR)+, metastatic) and MDA-MB-231 (ER+/PR+/HER2+, highly metastatic) were purchased from the American Type Culture Collection (ATCC, Manassas, VA, USA), and cultured according to the conditions recommended by ATCC. The cell lines were tested periodically and confirmed free from mycoplasma and/or cross-contamination with cells from different histological origin during laboratory manipulation or processing. All reagents were purchased from Invitrogen (Thermo Fisher Scientific, Waltham, MA, USA) unless otherwise specified. Cancer-associated fibroblasts (CAFs) were isolated from a breast cancer patient with written consent in adherence to Tri-Service General Hospital institutional research ethics committee IRB approved protocol (TSGH IRB No. 1–102–05-115), as previously described [15]. Briefly, tissues pulverized (Ø 1–2 mm) and disassociated in solution containing 400 IU collagenase I, 100 IU hyaluronidase, and 10% fetal bovine serum (FBS) were incubated at 37 °C overnight, centrifuged, and the fibroblasts-laden supernatant collected, centrifuged at 485×g for 30 min. The pellet containing the fibroblasts were then recovered and cultured in fibroblast growth medium consisting of Medium 199 and Ham’s F12 mixed 1:1 ratio, and 10% FBS. CAF was confirmed by the expression of alpha-smooth muscle actin (α-SMA), a biomarker for myofibroblasts. After sub-culturing 2–3 times, the CAFs were used for co-culture experiments where they were seeded in an insert, and cultured with either MDA-MB-231 or MCF7 breast cancer cells (at the bottom) in a Boyden chamber for further analyses. The co-culture assays without any anti-CDH11 antibody treatment spanned 72 h. For anti-CDH11 antibody treatment experiments, the metastatic breast cancer cells were harvested 72 h after co-culture with the CAFs and seeded into a fresh culture dish containing anti-CDH11 antibody for 48 h. Anti-CDH11 monoclonal antibody (clone 2C67, Cat. #.: H00001009-M25. Abnova Corporation, Taipei, Taiwan) and control IgG (Cat. #.: DAB14685-A01P) were used for cell culture at 1 mg/ml.

### Western blot assay

Protein from MCF7 and MDA-MB-231 parental or mammosphere-derived cells pre-exposed to different treatments, namely, anti-CDH11 antibody, miR-335 mimic, or miR-335 inhibitor, were loaded (20 μg/lane) and separated using 10% sodium dodecyl sulfate/polyacrylamide gel electrophoresis (SDS/PAGE), then the blots transferred onto polyvinylidene difluoride (PVDF) membranes, blocked in 5% skim milk in TRIS buffered saline (TBS) with Tween-20 (TBST) for 1 h, at room temperature, then incubated overnight at 4 °C with the primary antibodies against CDH11 (1:1000, clone 2C67, #H00001009-M25), E-cadherin (1:1000, clone 7H12, #MAB16359), vimentin (1:1000, clone 1G3, #H00007431-M10), b-catenin (1:2000, #MAB11143), FYN (1:1000, clone 3G11-F9, #H00002534-M01), EZH2 (1:1000, clone 2C3, #H00002146-M01), and b-actin (1:2000, clone 3G4-F9, #H00000060-M01) all purchased from Abnova (Abnova Corporation, Taipei, Taiwan). This was followed by incubation in peroxidase - conjugated secondary antibody for 1 h at room temperature, and washed with TBST three times. The signals were developed using enhanced chemiluminescence (ECL-Plus, Amersham Pharmacia Biotech, Piscataway, NJ, USA) and detected with a UVP BioSpectrum® Imaging System (Analytik Jena US LLC, Upland, CA, USA).

### Migration and invasion assays

For migration, a scratch-wound healing assay was performed as previously described [16]. The motility ratio (*MR*) was determined as:

$$ \left[\mathrm{denuded}\kern0.17em \mathrm{area}\;\mathrm{at}\;\mathrm{time}\hbox{'}x\hbox{'}\right]/\left[\mathrm{denuded}\kern0.17em \mathrm{area}\;\mathrm{at}\;\mathrm{time}\;0\right]. $$

For invasion assay, utilizing a 24-well plate matrigel Transwell® system, we seeded 3 × 10^4^ cells into the upper chamber of the insert (BD Bioscience, pore size = 8 μm) containing FBS-free medium, while the lower chamber contained medium supplemented with 10% FBS. After 24 h incubation, all media were discarded, non-invaded cells in the upper surface of the insert were carefully removed with sterile cotton bud, while invaded cells on the other side of the membrane were fixed with 3.7% formaldehyde and stained with crystal violet staining, and the average number of invaded cells were determined under microscope, from at least five randomly selected non-overlapping visual fields. The invasive ratio (*IR*) was calculated as:

$$ \left[\mathrm{number}\ \mathrm{of}\ \mathrm{invaded}\ \mathrm{cells}\right]/\left[\mathrm{total}\ \mathrm{number}\ \mathrm{of}\ \mathrm{cells}\right]\mathrm{number}\ \mathrm{of}\ \mathrm{invaded}\ \mathrm{cells} $$

### Mammosphere formation assay

For the generation of mammospheres, MCF7 or MDA-MB-231 cells (with or without treatments) were cultured under serum-deprived conditions using Corning® Costar® ultra-low attachment plates (Corning Inc., Corning, NY, USA). Briefly, 1 × 10^3^ cells were seeded per ml of mammosphere culture medium consisting of DMEM/F12, 2 mM L-glutamine, 100 U/ml penicillin, 100 U/ml streptomycin, 20 ng/ml recombinant human epidermal growth factor (hEGF, Sigma-Aldrich, St. Louis, MO, USA), 10 ng/ml recombinant human basic fibroblast growth factor (bFGF, Sigma-Aldrich, St. Louis, MO, USA) and B27 supplement (1×). After incubation for 7 days, the number and volume of mammospheres were scanned, analyzed and determined by a cell imager (Cell^3^iMager Neo, cc-3000, MiteK Lab Co., Ltd., New Taipei, Taiwan). The mammosphere formation efficiency was determined based on the average number of mammospheres formed. All experiments were performed four times in triplicates.

### Quantitative reverse transcription PCR (RT-qPCR) assays

All the reagents and kits for the total RNA extraction, cDNA synthesis, and detection of qPCR products were purchased from QIAGEN (QIAGEN Taipei, Taipei, Taiwan). 10 ng total RNA was used for reverse transcription using QIAGEN OneStep RT-PCR Kit (QIAGEN, Taiwan), and the polymerase chain reactions were performed using a Rotor-Gene SYBR® Green PCR Kit (400) (QIAGEN, Taiwan). The primer sequences for the qPCR assays are as listed in Additional file 2: Table S1. To analyze miR-335, hsa-miR-335_1 miScript Primer Assay was used, and performed according to the manufacturer’s instructions. For ectopic expression of miR-335, Syn-hsa-miR-335-5p miScript miRNA Mimic kit was used while Anti-hsa-miR-335-5p miScript miRNA Inhibitor was used for suppressing miR-335 expression. The MCF7 or MDA-MB-231 cells (with or without CAFs) were transfected with the mimic or inhibitor strictly following manufacturer’s protocols.

### CDH11 silencing

siRNA-mediated CDH11 silencing was performed strictly as earlier described by our team [11].

### Flow cytometry analysis of CD44/CD24 cell surface expression

The co-culture of MCF7 or MDA-MB-231 cells with CAFs was performed according to previously established protocol [15]. 1 × 10^6^ MCF7 or MDA-MB-231 cells were incubated with 10 μl anti-CD44-PE and anti-CD24-FITC antibody (Invitrogen, Carlsbad, CA, USA) at room temperature for 30 min. The labeled cells were washed twice and re-suspended in 200 μl PBS, and then analyzed using BD Accuri™ C6 flow cytometer (BD Bioscience, San Jose, CA, USA).

### miR-335 firefly luciferase reporter assay

1 × 10^5^ MCF7 or MDA-MB-231 cells were seeded per well in 24-well plates 24 h before miR-335 transfection. The miR-335 mimic/inhibitor transfection was performed using the Invitrogen™ Lipofectamine® 2000 transfection reagent, according to the manufacturer’s instructions (Thermo Fisher Scientific Inc., Waltham, MA, USA). 48 h after the miR-335 reporter plasmid transfection, luciferase activity was determined and analyzed using a Steady-Glo® luciferase assay system (#E2510, Promega Corp., Genelabs Life Science Corp., Taipei, Taiwan).

### In vivo tumor xenograft assay and non-invasive bioluminescence imaging

The tumor xenograft assays were approved by the institutional research ethics committee and performed in strict adherence to the Animal Use Protocol # LAC-2014-0170 according to the U.S. National Institutes of Health *Guide for the Care and Use of Laboratory Animals*. 4–6 week old female NOD/SCID mice (*n* = 30, median weight = 300 ± 1.7 g) were purchased from the BioLASCO (BioLASCO Taiwan Co., Ltd. Taipei, Taiwan). Mice, randomly allocated to groups (*n* = 5/group) by picking numbers from a dish, were inoculated orthotopically with 1 × 10^6^ MDA-MB-231 cells in the inguinal mammary fat pad. For anti-CDH11 antibody experiments, intravenous (i.v.) treatment with 1 mg/kg monoclonal anti-CDH11 antibody 5 times per week was initiated day 8 post-tumor inoculation. For miR-335 experiments, when tumors became palpable (mean tumor volume ~ 100 mm^3^) on day 7 post-tumor inoculation, using the in vivo polyethylenimine (in vivo-jetPEI®, Ref.#201-10G, Polypus Transfection, New York, NY, USA) carrier or delivery medium, we established miR-335 (miR-NC, miR-335 mimic or miR-335 inhibitor)/in vivo-jetPEI® complexes, according to manufacturer’s instruction. Treatment with the miR-335/PEI complexes consisting of 10 μg miR-335 and 1.2 μl in 100 μl of 5% glucose in vivo-jetPEI® reagent per injection, was done by intratumoral injection 3 times per day, every 72 h (t.i.d, q72h). Tumor growth was monitored by in vivo bioluminescence imaging (IVIS200 imaging system, Caliper Life Sciences Inc., Hopkinton, MA, USA). All mice were housed at a temperature of 20 ± 2 °C, humidity of 53 ± 5%, with a 12 h light/ 12 h dark cycle (lights on at 7:00 am) under specific pathogen-free (SPF) conditions, fed with standard LabDiet 5001 rodent diet (LabDiet, St. Louis, MO, USA), and supplied with clean drinking water, ad libitum. Post-experiment, all animals were humanely sacrificed by cervical dislocation.

### Statistical analysis

All data are expressed as means ± standard error of mean (SEM) and are representative of experiments performed at least four times in triplicates. Statistical analyses were performed using IBM SPSS Statistics for Windows, Version 25.0 (IBM Corp. Released 2017, Armonk, NY: IBM Corp) and GraphPad Prism 7.0 (San Diego, CA, USA). One-way ANOVA was used for inter-group comparison. A *p-*value ≤0.05 was considered as statistically significant.

## Results

### CDH11 is an independent prognosticator of poor clinical outcome and a regulator of the metastatic phenotype in breast cancer

**Fig. 1:**
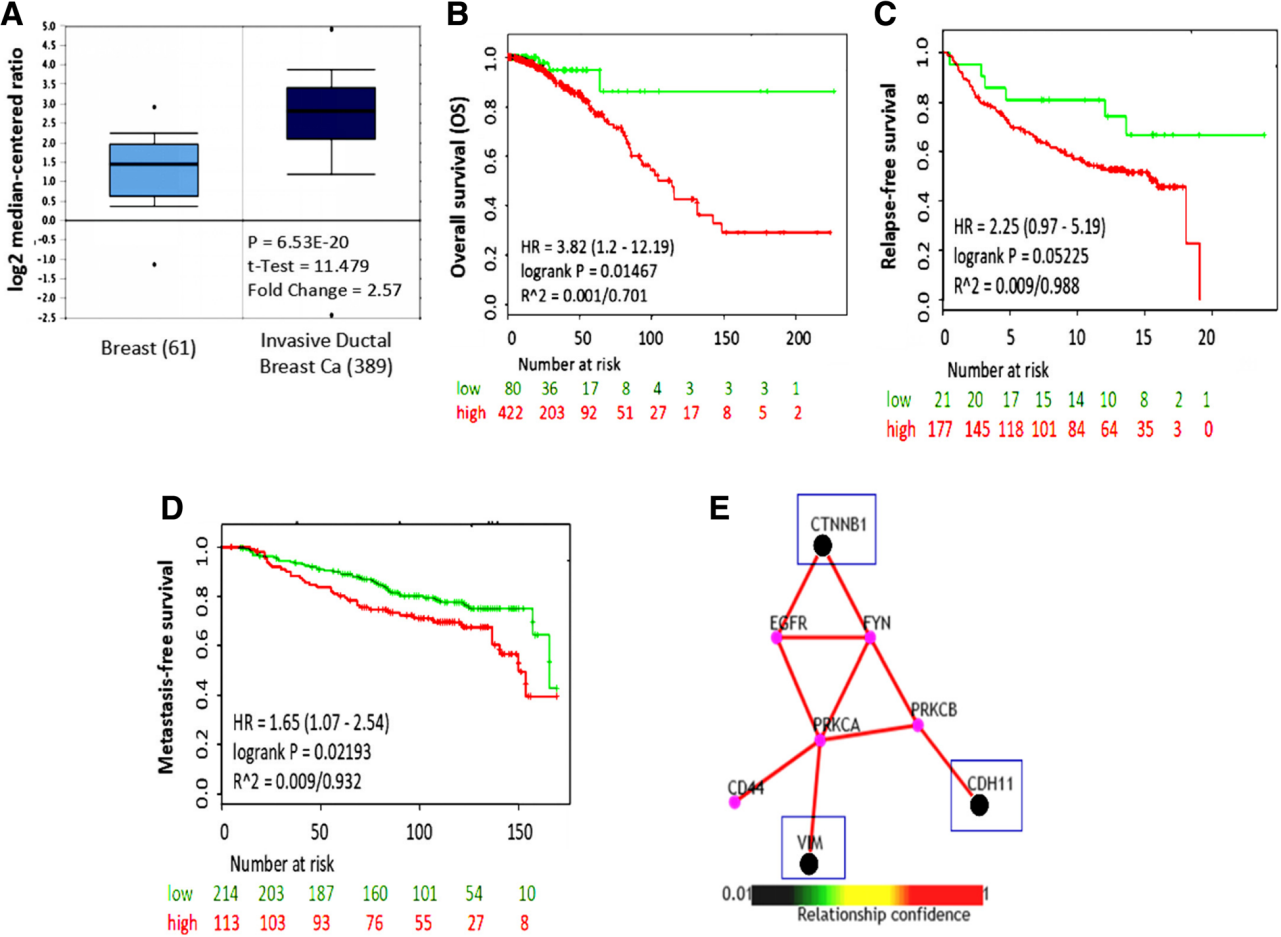
CDH11 is an independent prognosticator of poor clinical outcome and a regulator of the metastatic phenotype in breast cancer. **a** Analysis of GEO database showed that CDH11 mRNA level was approximately 2.5 folds higher in the invasive breast cancer cells (*N* = 389) than the normal breast tissues (*N* = 61). Kaplan-Meier plots showing (**b**) worse overall survival in patients with high CDH11 expression compared to the low expression group from analysis of TCGA breast invasive carcinoma cohort (*n* = 502), (**c**) high CDH expression confer shorter relapse-free survival, compared to the low expression group from the Desmedt-GSE7390 cohort (*n* = 198), and (**d**) the metastasis-free survival was worse in the high CDH11 group compared to the low group from the Kao Huang Breast GSE20685 cohort (*n* = 327). **e** PathwayNet network prediction of the functional connection of CDH11 with β-catenin, vimentin, FYN, CD44, EGFR, PRKCA and PRKCB

Understanding that distant metastasis is a precipitating cause of breast cancer-associated mortalities, identifying an actionable or druggable target for prevention or effective clinical management of advanced or metastatic breast cancer was the driving goal of the present study. Our bioinformatics-based gene expression profiling of The Cancer Genome Atlas (TCGA) invasive ductal breast carcinoma vs. normal breast cohort (*n* = 450) using the Oncomine platform [17] revealed that the CDH11 mRNA level was differentially over-expressed in the invasive (*n* = 389), compared to their non-invasive counterparts (*n* = 61) (2.6-fold increase, *p* = 6.53E-20; Fig. 1a); Furthermore, using the SurvExpress biomarker validation tool [18], our analysis of a normalized breast invasive carcinoma TCGA dataset (*n* = 502), revealed that compared to patients with low CDH11 expression, patients with high expression group exhibited worse overall survival with a survival disadvantage of 8.7, 40.5 and 67.9% at the 50th, 100th and 150th month, respectively with a concordance index of 53.96 and hazard ratio (HR) = 3.82 and confidence interval (CI) of 1.2–12.19 (*p* = 0.015; Fig. 1b). In parallel analyses, we also demonstrated that with a concordance index of 51.37 and HR = 2.25 (CI: 0.97–5.19), high CDH11 expression confers shorter relapse-free survival, compared to the low expression group using the Desmedt-GSE7390 cohort (*n* = 189) (*p* = 0.052; Fig. 1c), and the metastasis-free survival worse in the high CDH11 group compared to the low group from the Kao Huang Breast GSE20685 cohort (*n* = 327) with HR = 1.65 (CI = 1.07–2.54) (*p* = 0.022, Fig. 1d). To gain some mechanistic insight into the observed clinical effect of CDH11, using the PathwayNet [19], and we demonstrated that CDH11 interacts with and is functionally associated with key effectors of metastasis, namely, vimentin, CTNNB1(β-catenin), epidermal growth factor receptor (EGFR), FYN, protein kinase C alpha (PRKCA) and beta (PRKCB) types, and CD44 (Fig. 1e). These findings are suggestive of the role of CDH11 as an active regulator of metastasis in breast cancer.

### CAF-induced overexpression of CDH11 and the ensuing CDH11-enriched TME enhance the metastatic potential of breast cancer cells

**Fig. 2:**
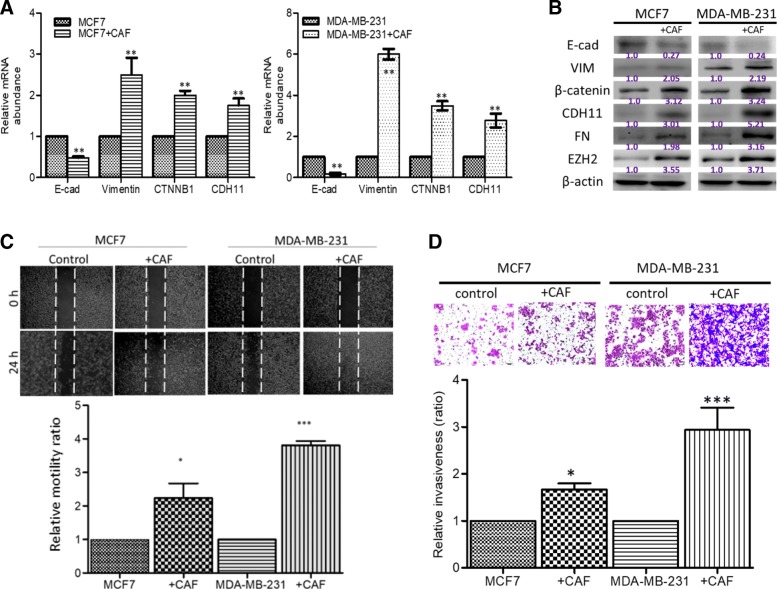
Cancer-associated fibroblasts (CAFs)-induced CDH11 overexpression promotes the metastatic potential of breast cancer cells. **a** Graphical representation of the differential expression of CDH11, vimentin and CTNNB1 in MCF7 (*left panel*) or MDA-MB-231 (*right panel*) cells, co-cultured with CAFs, compared to the independently cultured cells. **b** Photo-images with densitometry quantification from western blot analyses show the effect of CAF-co-culture on the expression of E-cadherin, CDH11, vimentin, β-catenin, FN, and EZH2 proteins in MCF7 or MDA-MB-231 cells. CDH11-enriched CAFs enhanced the (**c**) migratory and (**d**) invasive abilities MCF7 or MDA-MB-231 cells. CAF, cancer-associated fibroblast; CTNNB1, β-catenin; VIM, vimentin; +CAF, CAF co-cultured; FN, fibronectin; *p < 0.05; **p < 0.01; ***p < 0.001

Against the background that cancer-associated fibroblasts (CAFs) are CDH11-overexpressing and are important cellular components of the tumor microenvironment (TME) which plays a critical role in the induction or facilitation of tumor invasion and metastasis [20], we examined its role in breast oncogenicity and metastasis. Using a MCF + CAFs or MDA-MB-231 + CAFs co-culture system, while E-cadherin expression was markedly suppressed, we observed significantly co-upregulated expression of CDH11, vimentin, β-catenin, fibronectin (FN), and EZH2 in the human metastatic breast cancer cell lines, MCF7 and MDA-MB-231 co-cultured with CAFs, both at mRNA and protein levels compared to the independently cultured cells (*p* < 0.01; Fig. 2a and b). Consistent with the above, our scratch-wound healing assay, showed enhanced migration in the CDH11-enriched MCF7 + CAF (*p* < 0.05) or MDA-MB-231 + CAF (*p* < 0.001) cells, compared with their independent cell group (Fig. 2c). Similarly, the invasive capability was significantly enhanced in the CDH11-enriched MCF7 + CAF (*p* < 0.05) or MDA-MB-231 + CAF (*p* < 0.001) cells, compared with their independent cell group (Fig. 2d).

### Monospecific antibody targeting of CHD11 reduced the CD44^hi^CD24^neg/lo^ population and suppressed the metastatic potential of breast cancer cells

**Fig. 3:**
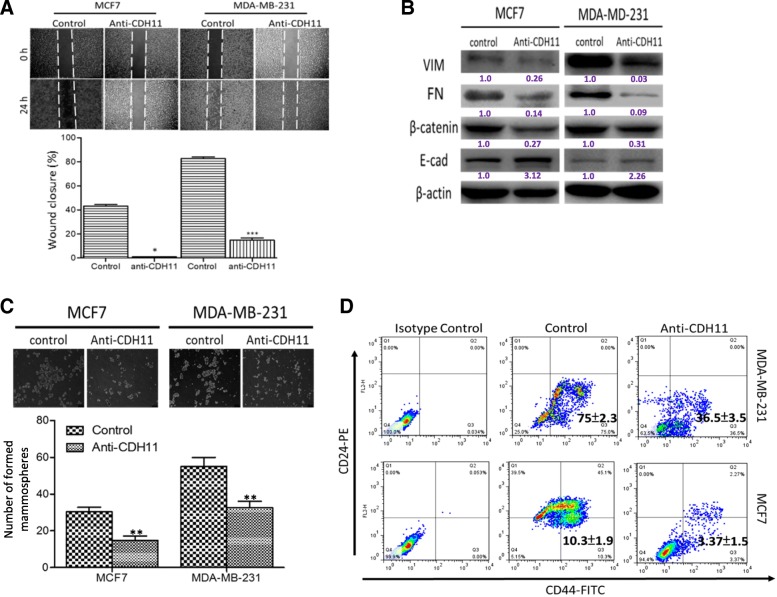
Monospecific antibody targeting of CHD11 reduced the CD44^hi^CD24^neg/lo^ population and suppressed the metastatic potential of breast cancer cells. **a** Photo-images (upper panel) and histograms (lower panel) show the effect of anti-CDH11 antibody treatment on the motility of MCF7 and MDA-MB-231 as demonstrated by scratch-wound healing assay. **b** Photo-images with densitometry quantification from western blot analyses show the effect of anti-CDH11 antibody treatment on the expression of vimentin, fibronectin, β-catenin, and E-cadherin proteins in MCF7 and MDA-MB-231 cells. **c** Photo-images (upper panel) and histograms (lower panel) show the effect of anti-CDH11 antibody on the quality and quantity of mammospheres formed. **d** Representative flow cytometry dot-plot of CD24-PE vs. *CD44-FITC* showing the effect of exposure to anti-CDH11 antibody on the CD44^hi^/CD24^neg/lo^ breast CSC population among the MCF7 and MDA-MB-231 cells. FITC, *fluorescein isothiocyanate; PE, phycoerytherin;* isotype control, FITC or PE conjugated IgG; *p ≤ 0.05, **p ≤ 0.01, ***p ≤ 0.001

Having established a role for CDH11 in the oncogenicity and progression of invasive breast cancer, we explored its therapeutic targetability of CDH11 and the probable anti-metastatic effect of same, using anti-CDH11 antibody. We demonstrated that compared to the untreated control, treatment with 1 mg/mL anti-CDH11 antibody significantly repressed the motility of the metastatic breast cancer cell lines, MCF7 (*p* < 0.05) and MDA-MB-231 (*p* < 0.001) (Fig. 3a). This finding was corroborated by marked down-regulation of the expression of metastasis-associated biomarkers, namely, vimentin, FN, and β-catenin, as well as moderately upregulated protein expression of E-cadherin (Fig. 3b). Since the self-renewal ability of metastatic cancerous cells has been associated to the EMT potential [21], and CDH11 implicated in both the metastatic and cancer stem cell-like phenotype of aggressive breast cancer [11] (also see Additional file 1: Figure S1), in parallel assays, we also showed that exposure to anti-CDH11 antibody elicited pronounced suppression of the mammosphere formation efficiency, as demonstrated by 2.2-fold (*p* < 0.01) and 1.9-fold (*p* < 0.01) decrease in the number of formed mammospheres, compared to the control group (Fig. 3c). In addition, flow cytometry analyses revealed that treatment with anti-CDH11 antibody elicited a ~ 2.0- and 3.0-fold reduction in the CD44^hi^/CD24^neg/lo^ breast CSC population, compared to their control counterparts (Fig. 3d).

### MicroRNA-335 mediates anti-CDH11 antibody therapy response and an enhanced miR-335/CDH11 ratio elicits marked suppression of the CSC-like and metastatic phenotypes of invasive breast cancer

**Fig. 4:**
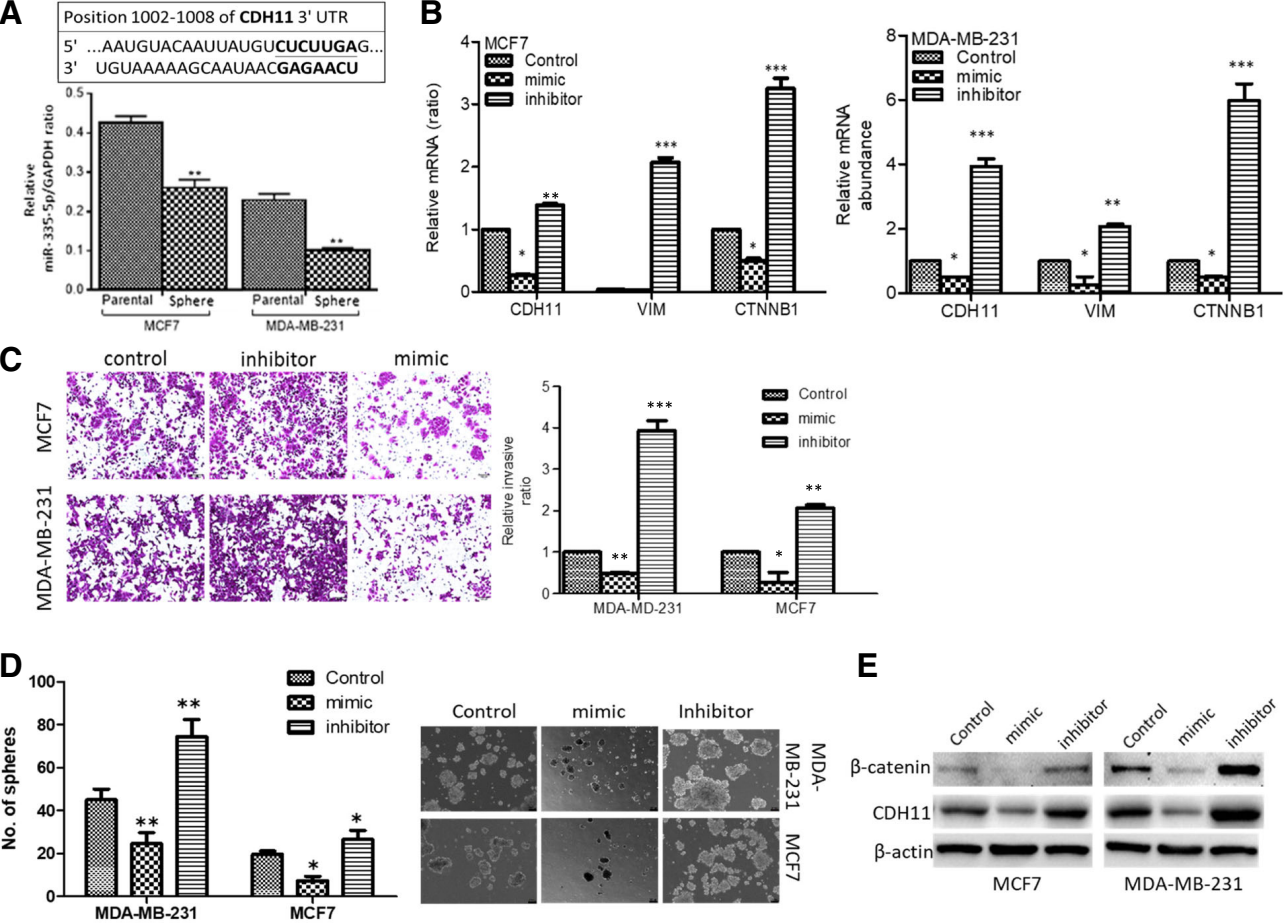
MiR-335 directly targets CDH11 and reverses CDH11-induced cancer-related biological activities. **a** Graphical representation of the differential expression of miR-335 in parental MCF7 or MDA-MB-231 cells and their mammospheres. **b** Histograms showing the effect of miR-335 mimic or inhibitor on the mRNA expression of CDH11, vimentin and β-catenin in MCF7 or MDA-MB-231 cells. **c** Photo-images (*left panel*) and histograms (*right panel*) showing the effect of miR-335 mimic or inhibitor on the mRNA expression of CDH11, vimentin and β-catenin in MCF7 or MDA-MB-231 cells, compared to control. **d** The effect of miR-335 mimic or inhibitor on mammosphere formation, compared to the control group. **e** Western blot photo-images of the effect of miR-335 mimic or inhibitor on the protein expression of CDH11 and β-catenin, compared to control. *p < 0.05; **p < 0.01; ***p < 0.001

In a bid to elucidate the underlying mechanism for the observed effects of the altered expression of CDH11 in invasive breast cancer, we tested the hypothesis that CDH11-enhanced CSC-like and metastatic phenotype is inversely correlated with the expression of miR-335 in invasive breast cancer, especially as Sandoval-Bórquez A, et al. [14] recently suggested that elevated levels of CDH11 in gastric cancer were associated with low expression of miR-335. Using the specified 3’UTR binding site of *CDH11* for miR-335, we observed that cells derived from MCF7 and MDA-MB-231 mammospheres exhibited lower expression of miR-335 mRNA compared to their parental counterpart (Fig. 4a). In addition, we examined the therapeutic implication of this miR-335/CDH11 ratio in the metastatic MCF7 and MDA-MB-231 cells using miR-335 mimic and inhibitor. We demonstrated that exposure to miR-335 mimic significantly repressed miR-335 inhibitor-induced increase in CDH11, vimentin and β-catenin mRNA expression levels in the human metastatic MCF7 and MDA-MB-231 cells (Fig. 4b).

In parallel assays, we also showed that re-expression of miR-335 using miR-335 mimic, markedly suppress the ability of the metastatic MCF7 or MDA-MB-231 cells to invade, compared to their untreated control or inhibitor-treated counterparts (Fig. 4c). Similarly, while the miR-335 mimic-induced re-expression of miR-335 markedly suppress the ability of the metastatic MCF7 or MDA-MB-231 cells to form mammospheres, compared to their untreated control or inhibitor-treated counterparts, miR-335 inhibitor-induced reduction in miR-335 levels, resulted in the restoration of mammosphere formation ability in the metastatic breast cell lines (Fig. 4d). In addition, we showed that these bio-phenomena were associated with miR-335 mimic-induced suppression of CDH11 and β-catenin protein expression levels and converse upregulated CDH11 and β-catenin expression by miR-335 inhibitor, compared to the control group in the metastatic MCF7 or MDA-MB-231 cells (Fig. 4e).

### Treatment with anti-CDH11 antibody or miR-335 mimic markedly suppressed breast cancer metastasis, in vivo and ex vivo

**Fig. 5:**
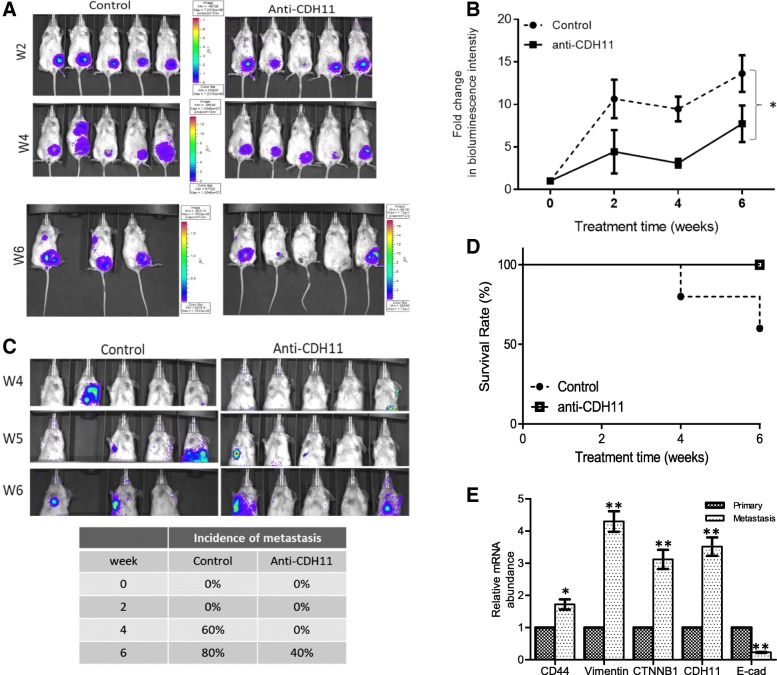
Anti-CDH11 antibody treatment suppressed tumorigenesis and cancer metastasis, in vivo. **a** Non-invasive bioluminescence imaging of xenograft mice show the effect of anti-CDH11 antibody treatment on tumor burden, compared to control group. **b** ‘Tumor burden versus time’ curve depicting the fold change in tumor burden-related bioluminescence over time; *p ≤ 0.05 (**c**) The effect of anti-CDH11 antibody on distant metastasis incidence in vivo, as shown by bioluminescence imaging. The metastasis incidence is summarized in the Table. **d** Kaplan-Meier survival curve of the effect of anti-CDH11 antibody on the survival rate of xenograft mice after 6-week experimental period. **e** Comparative qPCR analysis of CDH11, CD44, vimentin, CTNNB1 and E-cadherin mRNA expression in the primary versus metastatic tumor samples from the control mice. Both primary and metastatic tumor tissues were harvested from the mice at the end of the experiment. The metastatic tumors were mainly harvested from the lungs. W, week post-inoculation; *p < 0.05; **p < 0.01; ***p < 0.001

**Fig. 6:**
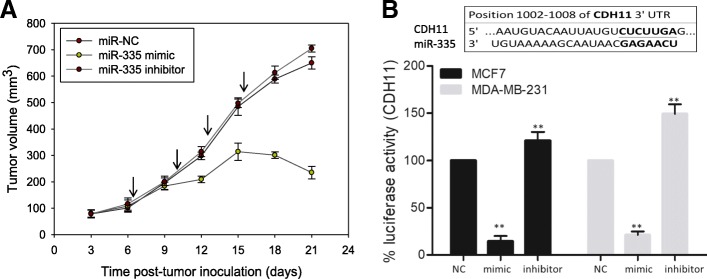
Intratumoral injection of miR-335 suppresses tumor growth and regulates CDH11 expression by targeting its promoter 3’UTR site in xenografts. **a** Graph showing the effect of intratumoral injection of miR-NC, miR-335 mimic or miR-335 inhibitor in complex with in vivo-jetPEI at the arrow-indicated time points on tumor growth. **b** Graphical representation of the effect of miR-NC, miR-335 mimic or miR-335 inhibitor on CDH11 luciferase activity by targeting the specified target sites, as determined by luciferase reporter assays in MCF7 or MDA-MB-231 cells. *p < 0.05; **p < 0.01; ***p < 0.001

After establishing the role of CDH11 role as an actionable inductor and/or modulator of metastasis and stemness in vitro, we utilized the murine tumor xenograft model for in vivo validative studies. After inoculation with MDA-MB-231 or MCF cells, the tumor-bearing mice were randomly divided into control and anti-CDH11 groups. In vivo non-invasive imaging showed that treatment with the anti-CDH11 antibody significantly inhibited tumor growth in a time-dependent manner, so that by week 6 of treatment, mice injected with anti-CDH11 antibody exhibited significantly lower tumor burden as compared to their control counterparts, as reflected by the intensity of the bioluminescence; in fact of the 5 mice bearing tumors, 3 had apparently lost tumor-associated bioluminescence in the anti-CDH11 group by week 6, while for the control group, 2 had died secondary to life-incompatible tumor size and/or metastases, and there was progressive expansion of tumor bioluminescence in the 3 still alive (Fig. 5a). The tumor burden curve representing the change in the bioluminescence over time demonstrated that the tumor burden in the anti-CDH11 group was significantly lower with 37.1, 30.6, and 57.7% lesser tumor bioluminescence intensity in the anti-CDH11 mice by week 2, 4, and 6 or treatment, respectively, compared to the control mice (*p* < 0.05) (Fig. 5b). Interestingly, by week 4 of treatment, first incidence of metastasis (lung) was observed in one of the control mice; the same mouse died by week 5 while two more different incidences of metastases occurred. By week 6, metastases were observed in 4 of the 5 mice in the control group, and 2 had died (Fig. 5c). For the anti-CDH11 mice, the first incidence of metastasis observed was at the end of week 5 (1 week later than the control). By the end of the 6-week animal study, only 3 mice survived in the control group while all the mice from the anti-CDH11 group survived; thus, anti-CDH11 conferred a 40% survival advantage and reduced the metastasis index by 40% (Fig. 5c and d). All mice were free from bacterial, viral, and parasitic pathogens listed in the Taiwan guideline for care and use of laboratory animals, with no dyscrasia, nor marked difference in body weight observed between experimental groups before and after the experiments. In addition, ex vivo, we compared the CDH11 expression between the primary and metastatic tumors from the xenograft mice. CDH11 mRNA was significantly higher in the metastatic tumor samples as compared to their primary tumor counterparts, and this was concomitant with down-regulated E-cadherin (*p* < 0.01) and upregulated CD44 (p < 0.05), vimentin (*p* < 0.01), and β-catenin (*p* < 0.01) mRNA expression in the metastasis sample, compared to primary tumor samples (Fig. 5e). In parallel validation in vivo studies, breast tumor-bearing mice were injected with miR-335-NC, mimic, or inhibitor in complex with the in vivo-jetPEI delivery medium, q72h, *t.i.d*. By day 18 post-tumor inoculations (2 day after the fourth round of miR-335 injections), tumor growth was significantly suppressed in the miR-335 mimic mice, compared to the miR-NC or miR-335 inhibitor mice (*p* < 0.01), and a decline in tumor volume was appreciable (Fig. 6a). In addition, using the firefly luciferase assay, we demonstrated that the observed therapeutic effect of anti-CDH11 antibody is through the direct targeting of CDH11 3’UTR promoter sites by the upregulated miR-335 (Fig. 6b). Taken together, these results are indicative of the CDH11-targeting role miR-335, in vivo, and suggestive of its use as a therapeutic strategy with promising efficacy.

## Discussion

Despite improved cancer survival rates in the last decade, cancer metastasis, accounting for ~ 90% of cancer-related deaths, remains a principal cause of cancer progression and mortality, exposing the progress-lag in the clinical management of metastatic or advanced stage disease. This may not be unconnected with the excessive emphasis on tumor growth inhibition at the expense of metastasis prophylaxis and/or treatment. Consistent with increasing understanding of the enigmatic tumor niche and multi-factorial metastatic phenotype, as well as evolving treatment paradigms for metastatic breast cancer, the present study is based on the premise that concomitant inhibition of both cancer growth and metastasis is clinically feasible and that it is in itself an essential step to elicit good therapeutic response and clinical outcomes.

In the present study, for the first time, to the best of our knowledge, we demonstrated that CDH11 is an independent prognosticator of poor clinical outcome and a regulator of the metastatic phenotype in invasive breast cancer (Fig. 1). This is clinically-relevant and consistent with recent report from weighted gene co-expression network analysis by Chen PF, et al. [22], that CDH11 is aberrantly expressed in gastric cancer, and exhibits a strong positive correlation with gastric cancer TNM stage than observed in other hub genes; with survival analysis associating poor prognosis with gastric cancer samples with high CDH11 expression. This is also corroboratory of our recently published work wherein we suggested a correlation between high CDH11 Expression and the poor overall survival rates in patients with basal-like and triple negative breast cancer based on big data analysis [11]. With the understanding that CAFs are CDH11-overexpressing and are important active cellular components of the tumor niche which invariably plays a critical role in the induction or facilitation of tumor invasion and metastasis [20], we examined its role in breast oncogenicity and metastasis, and demonstrated that CAF-induced overexpression of CDH11 and the ensuing CDH11-enriched TME enhance the metastatic potential of breast cancer cells (Fig. 2). Working on the premise that establishing a causative, or more appropriately, inductive and facilitative association between CDH11 overexpression and cancer metastasis, is a vital step towards tagging it a targetable or actionable molecular driver of oncogenicity, and defining its therapeutic relevance in the effective management of advanced stage or metastatic breast cancer, we also provided preclinical evidence that the monospecific antibody targeting of CHD11 reduced the CD44^hi^CD24^neg/lo^ population and suppressed the metastatic potential of breast cancer cells (Fig. 3). Firstly, this accentuates the role of CDH11 in cancer progression secondary to its induction of EMT in a tumor niche that is permissive for acquisition of migratory, invasive and CSC-like phenotypes, thus, highlighting a role for CDH11 as a vital molecular link at the interphase between EMT and cancer stemness. Secondly, this is of translational significance especially in the context of accruing evidence on the functional and phenotypic association between EMT, a critical step in metastasis, and cancer stemness [21, 23, 24]. For instance, it has been shown that the ectopic expression of Snail1/Twist1 in breast epithelial cells or their exposure to oncogenic signaling such as TGF-β induces a phenotypically stem cell-like population with enhanced CD44 (CD44high) and low CD24 (CD24low) expression, as well as the ability to form mammospheres [21]; therefore, it is not out of place that the treatment of human metastatic breast cancer cell lines, MCF7 and MDA-MB-231 with monospecific antiCDH11 antibody reduced the CD44^hi^CD24^neg/lo^ population, suppressed their motility and mammosphere formation efficiency, as well as elicited the repression of metastasis-related EMT markers vimentin, fibronectin and β-catenin, while up-regulating E-cadherin (Fig. 3). Since standard chemotherapeutics show little efficacy in treatment of advanced stage or metastatic breast cancer, our use of antiCDH11 monoclonal antibody is reminiscence of the need for smart chemotherapeutics and aligns with emerging anticancer treatment strategies that use biotherapies that interfere with novel targets and signaling pathways [25, 26]. This is also corroborated by a previous study demonstrating that anti-CDH11 antibody effectively suppressed bone metastasis in prostate cancer mouse model [27].

To give mechanistic insight into the therapeutic effect of antiCDH11 antibody treatment observed in metastatic breast cancer cells, we further demonstrated that miR-335 mediates anti-CDH11 antibody therapy response and that an enhanced miR-335/CDH11 ratio elicits marked suppression of the CSC-like and metastatic phenotypes of invasive breast cancer (Fig. 4), thus revealing a therapeutically-exploitable inverse correlation between CDH11-enhanced CSC-like and metastatic phenotype and the expression of miR-335 in invasive breast cancer. Our finding is consistent with that of Sandoval-Bórquez A, et al. [14] that recently showed that elevated levels of CDH11 in gastric cancer were associated with low expression of miR-335, suggesting a role for miR-335-5p as a potential suppressor of metastasis and invasion in gastric cancer.

More interestingly, the antimetastasis cum anti-CSC therapeutic effects elicited by the antibody-mediated specific targeting of CDH11 via upregulated miR-335 activity observed in vitro were validated in vivo as reflected in our preclinical demonstration of how treatment with anti-CDH11 antibody or miR-335 mimic markedly suppressed breast cancer metastasis, in vivo and ex vivo (Fig. 5). This is important, since EMT as a biological process has been linked with the acquisition of CSC-like properties, where CSCs are implicated in the initiation, maintenance and propagation of cancerous cells, as well as mediate resistance to anticancer therapy [21, 23, 24]; thus by targeting CDH11, we triggered re-expression of tumor suppressor miR-335, which curtailed CDH11-induced EMT, consequently repressing CSC activities, enhancing therapeutic response and improving clinical outcome.

## Conclusion

In this study, we highlight the role of CDH11 in invasive breast cancer metastatic and CSC-like phenotype, as well as showcase the miR-335-mediated therapeutic efficacy of anti-CDH11 antibody treatment. Mechanistically, we showed that akin to a biological negative feedback loop, upregulated miR-335 targets and suppresses CDH11 expression and/or activity as well as dampen the erstwhile strong CDH11-induced EMT/stemness signaling. Finally, our results further beg the case for the clinical application of monospecific anti-CDH11 antibody as a therapeutic option for treatment of patients with metastatic breast cancer. However, further development of humanized anti-CDH11 will be required for clinical use.

## Additional files

Additional file 1: **Figure S1.** Silencing CDH11 suppresses ALDH1 expression. (A) Photo-image of the effect of siRNA-mediated loss of CDH11 function on CDHH11 and ALDH1 protein expression levels in MCF7 or MDA-MB-231 cells, as shown by western blot analysis. (B) Graphical representation of A. Results represent mean ± SD of 3 independent assays in triplicate. * *p* < 0.05, ** *p* < 0.01, *** *p* < 0.001. (PPTX 82 kb)

Additional file 2: **Table S1.** Primers used for RT-qPCR in this study. **Figure S1.** Luciferase reporter assays. Human CDH11 3’UTR luciferase plasmid was transfected into both MCF7 and MDA-MB-231 cells for the test. The luciferase activity is a measurement of cdh11 mRNA transcripts. When miR-335 mimic molecules were transfected, the luciferase activity was significantly reduced while the opposite was true for the miR-335 inhibitor. Both MCF7 and MDA-MB-231 cells demonstrated the similar phenomenon. **p < 0.01. **Figure S2.** Mir-335 restoration suppressed EMT markers in metastatic cancer cells. Data was obtained from database GSE9586 [27] and demonstrated that CDH11 mRNA level was prominently elevated in the metastatic breast cancer cells along with vimentin, CTNNB1 (β-catenin) as compared to E-cad (CDH1). When miR-335 level was restored, the mRNA level of CDH11, vimentin, CTNNB1 was significantly reduced while E-cad (CDH1) was increased. *p < 0.05; **p < 0.01. (DOCX 267 kb)

## Data Availability

All data generated or analyzed during this study are included in this published article [and its supplementary information files]. Please contact the corresponding author for additional information or supporting data

## Abbreviations

Ad libitum: as desired
CAF: Cancer-associated fibroblast
CD: Cluster of differentiation
CDH11: Cadherin 11
CSC: Cancer stem cell
EGFR: Epidermal growth factor receptor
EMT: Epithelial-to-mesenchymal transition
EZH2: Enhancer of zeste homolog 2
FN: Fibroblast
hi: High
lo: Low
miR: MicroRNA
NC: Negative control
neg: Negative
NOD/SCID: Non-obese diabetic severely combined
PRKCA: Protein kinase C alpha type immunodeficiency
PRKCB: Protein kinase C alpha type
*t.i.d.*: *Ter in die*, 3 times a day
TGF-β: Tumor growth factor beta protein
TME: Tumor microenvironment
TNM: Tumor, node, and metastasis
UTR: Untranslated region
