# Supplementary figures and images for: Monospecific antibody targeting of CDH11 inhibits epithelial-to-mesenchymal transition and represses cancer stem cell-like phenotype by up-regulating miR-335 in metastatic breast cancer, in vitro and in vivo
Source: BMC Cancer. 2019 Jun 27;19:634. doi: 10.1186/s12885-019-5811-1 (PMC6598338; doi:10.1186/s12885-019-5811-1)

## Slide 1
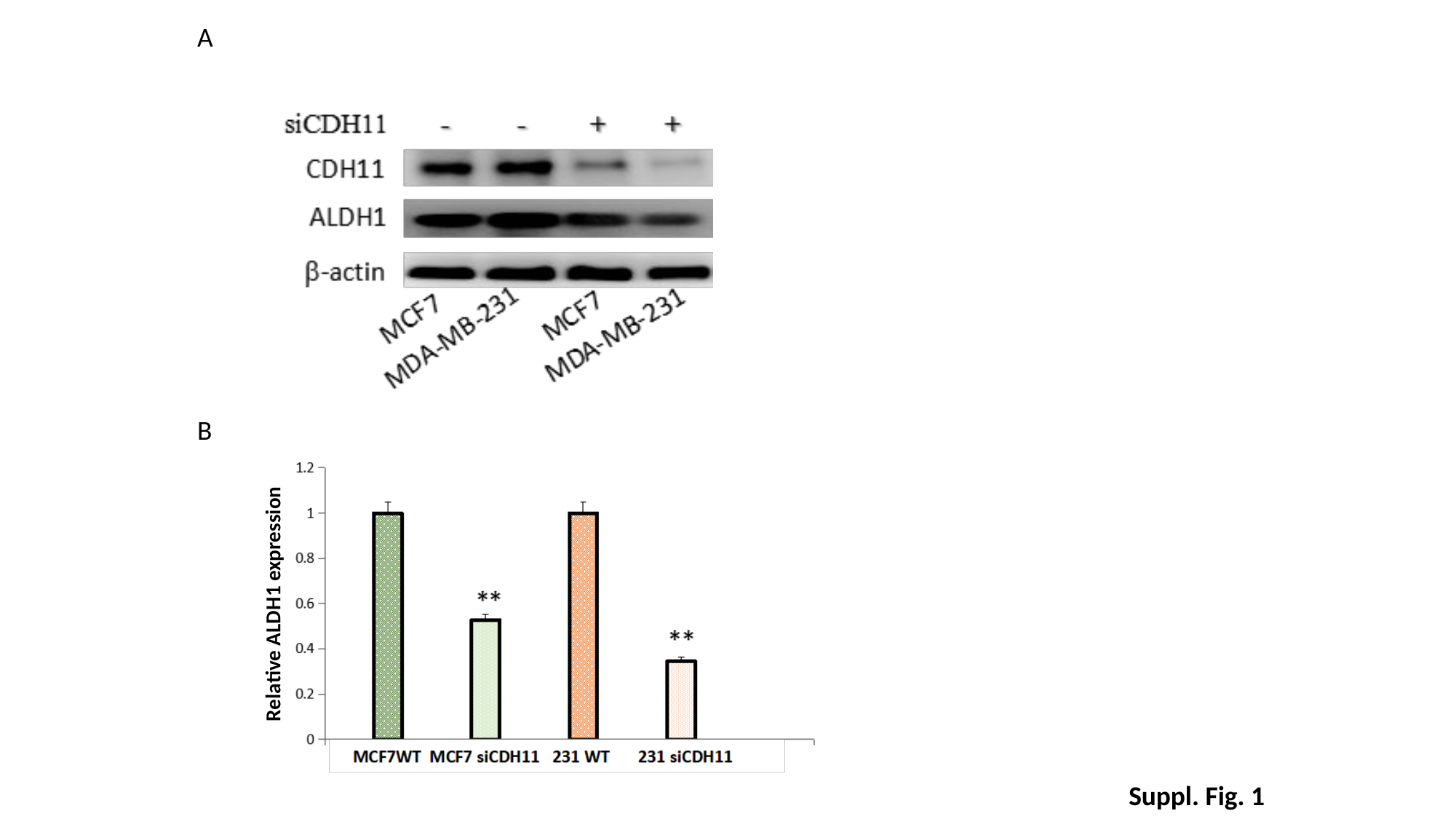

A
B
Relative ALDH1 expression
Suppl. Fig. 1

Supplement: Supplementary file 1 — Figure S1. Silencing CDH11 suppresses ALDH1 expression. (A) Photo-image of the effect of siRNA-mediated loss of CDH11 function on CDHH11 and ALDH1 protein expression levels in MCF7 or MDA-MB-231 cells, as shown by western blot analysis. (B) Graphical representation of A. Results represent mean ± SD of 3 independent assays in triplicate. * p < 0.05, ** p < 0.01, *** p < 0.001. (PPTX 82 kb) [file 12885_2019_5811_MOESM1_ESM.pptx]
