# Supplementary material for: Monospecific antibody targeting of CDH11 inhibits epithelial-to-mesenchymal transition and represses cancer stem cell-like phenotype by up-regulating miR-335 in metastatic breast cancer, in vitro and in vivo
Source: BMC Cancer. 2019 Jun 27;19:634. doi: 10.1186/s12885-019-5811-1 (PMC6598338; doi:10.1186/s12885-019-5811-1)
Supplement: Supplementary file 2 — Table S1. Primers used for RT-qPCR in this study. Figure S1. Luciferase reporter assays. Human CDH11 3’UTR luciferase plasmid was transfected into both MCF7 and MDA-MB-231 cells for the test. The luciferase activity is a measurement of cdh11 mRNA transcripts. When miR-335 mimic molecules were transfected, the luciferase activity was significantly reduced while the opposite was true for the miR-335 inhibitor. Both MCF7 and MDA-MB-231 cells demonstrated the similar phenomenon. **p < 0.01. Figure S2. Mir-335 restoration suppressed EMT markers in metastatic cancer cells. Data was obtained from database GSE9586 [27] and demonstrated that CDH11 mRNA level was prominently elevated in the metastatic breast cancer cells along with vimentin, CTNNB1 (β-catenin) as compared to E-cad (CDH1). When miR-335 level was restored, the mRNA level of CDH11, vimentin, CTNNB1 was significantly reduced while E-cad (CDH1) was increased. *p < 0.05; **p < 0.01. (DOCX 267 kb) [file 12885_2019_5811_MOESM2_ESM.docx]

**Supplementary Data**

**Supplementary Table 1** Primers used fo**r** RT-qPCR in this study.

**Table S1. Primers used for RT-qPCR**

| **Gene name** | **Forward Primer Sequence (5’-3’)** | **Reverse Primer Sequence (5’-3’)** |
| --- | --- | --- |
| *Vimentin* | TCTCTGAGGCTGCCAACCG | CGAAGGTGACGAGCCATTTCC |
| *CDH11* | TGGCAGCAAGTATCCAATGG | TTTGGTTACGTGGTAGGCAC |
| *E-cadherin* | CAGAAAGTTTTCCACCAAAG | ACTGAACCTGACCGTACA |
| CTNNB1 | TTGATGGAGTTGGACATG | CAGCTACTTGTTCTTGAG |

**Supplementary Figure 1** Luciferase reporter assays. Human CDH11 3’UTR luciferase plasmid was transfected into both MCF7 and MDA-MB-231 cells for the test. The luciferase activity is a measurement of cdh11 mRNA transcripts. When miR-335 mimic molecules were transfected, the luciferase activity was significantly reduced while the opposite was true for the miR-335 inhibitor. Both MCF7 and MDA-MB-231 cells demonstrated the similar phenomenon. ***p*<0.01

**
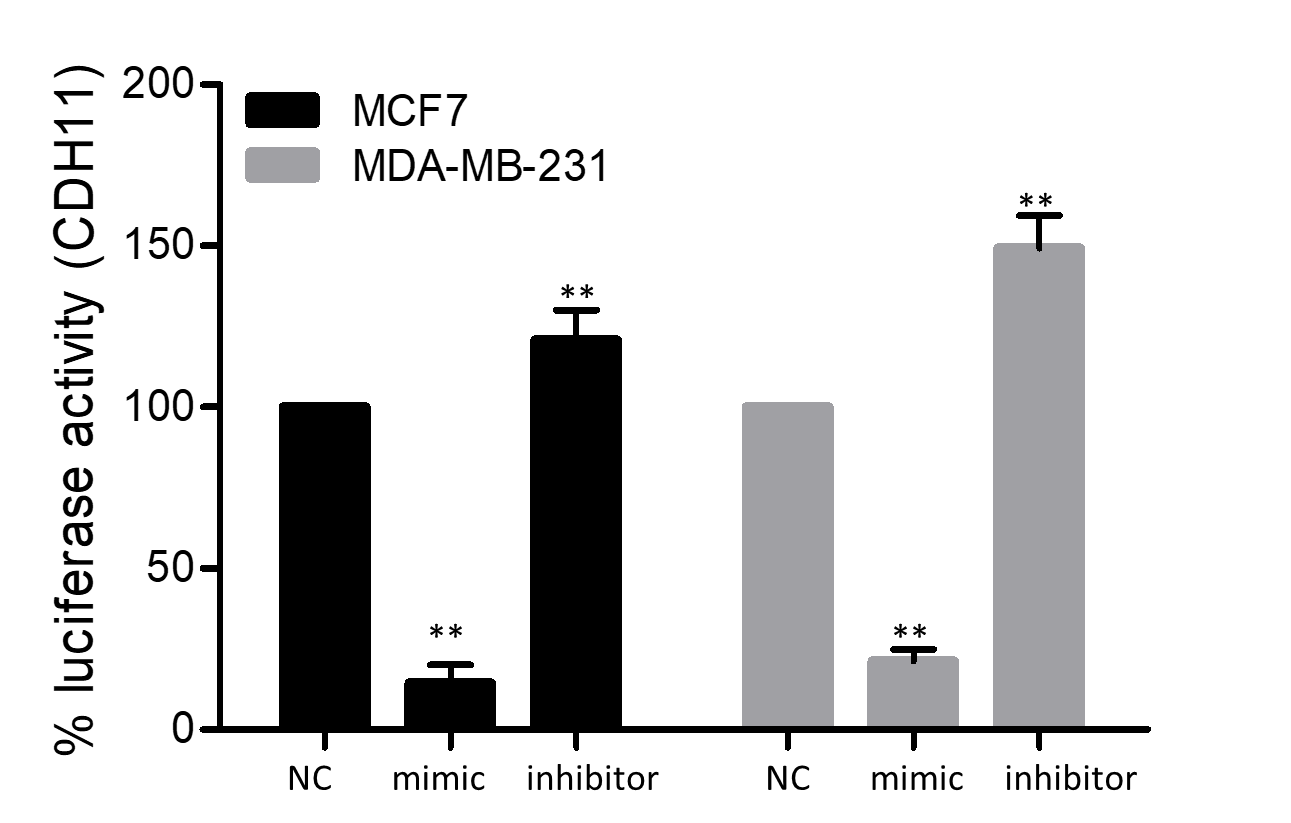
**

**Supplementary Figure 2.** Mir-335 restoration suppressed EMT markers in metastatic cancer cells. Data was obtained from database GSE9586 [27] and demonstrated that CDH11 mRNA level was prominently elevated in the metastatic breast cancer cells along with vimentin, CTNNB1 (β-catenin) as compared to E-cad (CDH1). When miR-335 level was restored, the mRNA level of CDH11, vimentin, CTNNB1 was significantly reduced while E-cad (CDH1) was increased. *p<0.05; **p<0.01.


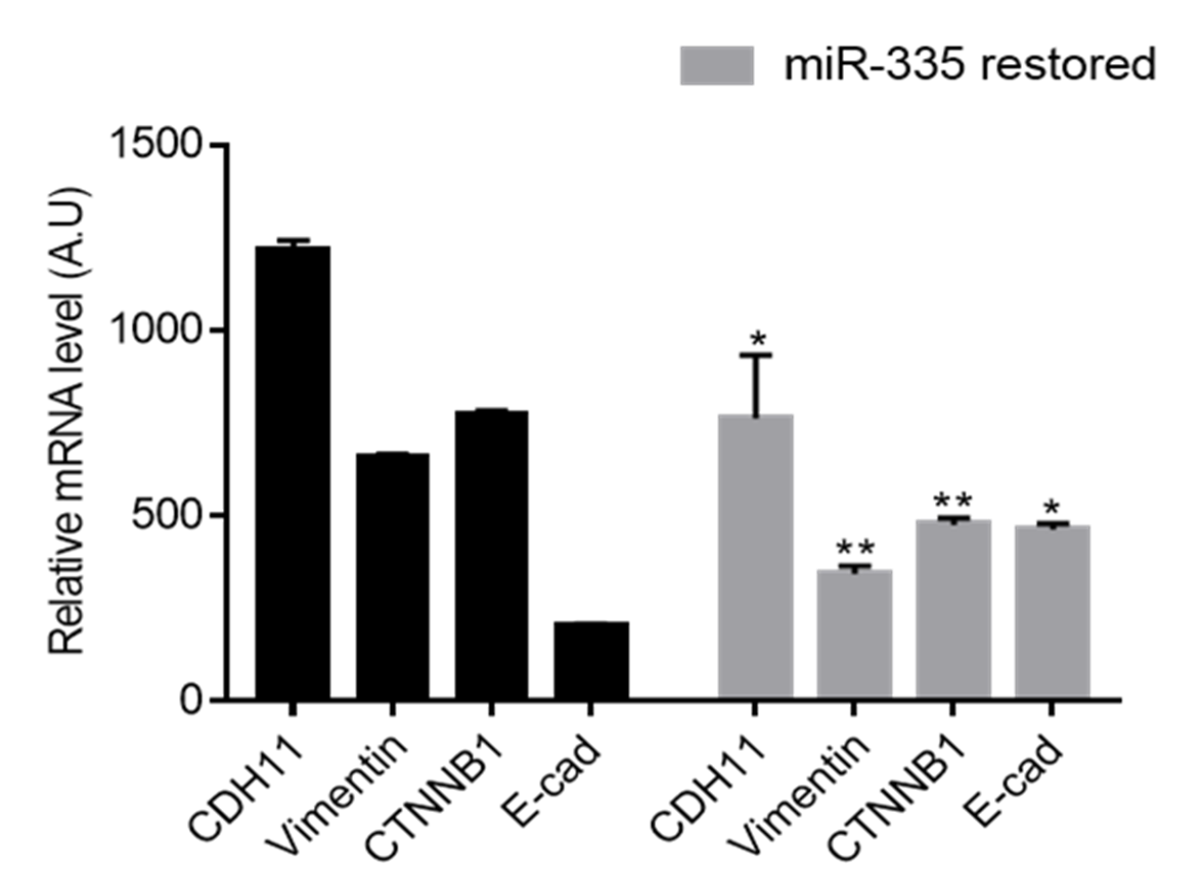


**miR-335 suppressed miR-335 restored**
